# Coexistence of multiple anti-neuronal antibodies in autoimmune encephalitis in China: A multi-center study

**DOI:** 10.3389/fimmu.2022.858766

**Published:** 2022-09-23

**Authors:** Shan Qiao, Shan-Chao Zhang, Zhi-Hao Wang, Lei Wang, Ran-Ran Zhang, Hai-Yun Li, Yang Jin, Ling-Ling Liu, Mei-Ling Wang, Ai-Hua Wang, Xue-Wu Liu

**Affiliations:** ^1^ Department of Neurology, The First Affiliated Hospital of Shandong First Medical University & Shandong Provincial Qianfoshan Hospital, Jinan, China; ^2^ Department of Medical Genetics, School of Basic Medical Sciences, Cheeloo College of Medicine, Shandong University, Jinan, China; ^3^ School of Medicine, Cheeloo College of Medicine, Shandong University, Jinan, China; ^4^ Department of Neurology, Qilu Hospital of Shandong University, Jinan, China; ^5^ Department of Neurology, Liaocheng People’s Hospital, Liaocheng, China; ^6^ Department of Neurology, Binzhou Medical University Hospital, Binzhou, China; ^7^ Institute of Epilepsy, Shandong University, Jinan, China

**Keywords:** autoimmune encephalitis, antibodies coexistence, multiple anti-neuronal antibodies, prognosis, lung cancer

## Abstract

**Background:**

Given that the combination of multiple antibodies in autoimmune encephalitis (AE) is rare and its clinical significance is unclear, this study aimed to investigate the clinical characteristics and significance of overlapping multiple anti-neuronal antibodies in patients with AE.

**Methods:**

We conducted a retrospective analysis of the clinical characteristics, treatment, and prognostic details of 22 patients with multiple coexisting antibodies from multiple clinical centers in China.

**Results:**

Among the 276 patients who were AE antibody-positive, 22 (7.97%) had two or more antibodies. Among the 22 patients with coexisting AE-related antibodies, 14 patients (63.63%) were combined of cell surface and intracellular antibody, and the remaining 8 patients (36.36%) were detected to be cell surface antibody positive only. The main symptoms of the 22 patients in this cohort included fever, seizures, memory impairment, cognitive decline, and sleep disorders. Five (22.73%) patients had tumors, among whom four had small-cell lung cancers, and one had mediastinal tumors. A total of 20 patients were treated with steroids and intravenous immunoglobulin, and 18 showed varying degrees of symptomatic improvement after first-line immunotherapy. Three patients died of tumor progression or chemotherapy complications.

**Conclusion:**

The coexistence of multiple anti-neuronal antibodies in patients with AE may cause a superimposition and diversification of clinical manifestations. Combined paraneoplastic antibody positivity may be suggestive of an underlying malignancy.

## Introduction

Autoimmune encephalitis (AE) is an uncommon neurological disorder mediated by autoimmune mechanisms and is an important cause of rapidly progressive cognitive dysfunction, refractory epilepsy, and psychiatric abnormalities (1, 2). While most patients have been shown to respond well to immunotherapy, some patients continue to have intractable seizures and varying degrees of cognitive impairment, which have a serious effect on their quality of life (3, 4).

With the development of detection technology, an increasing number of AE antibodies have been reported (5–7). AE-associated antibodies can be divided into two categories: antibodies against cell-surface-targeting antigens and antibodies against intracellular-targeting antigens. The cell surface-targeted antigen antibodies comprise primarily the anti-N-methyl-D-aspartate receptor (NMDAR) antibodies, anti-g-aminobutyric acid B receptor (GABABR) antibodies, anti-glioma inactivated 1 protein (LGI1) antibodies, anti-contactin-associated protein-like 2 (CASPR2) antibodies, and anti-AMPAR antibodies. Intracellular antibodies primarily include the anti-Hu, Yo, Ri, Ma2, CV2/CRMP5, amphiphysin, and glutamic acid decarboxylase (GAD) antibodies. The clinical manifestations and prognoses of the various AE subtypes differ (6, 8). Given of the diversity of the antibody subtypes and the clinical manifestations of AE as well as the disorder’s insidious onset in some patients (e.g., minor cognitive dysfunction), early diagnosis and treatment of AE remain difficult. Therefore, an in-depth study of the clinical features, treatment, and prognosis of the different antibody subtypes associated with AE is of great significance.

In recent years, investigators have shown the presence of co-existent neuronal antibodies in patients with AE (9–11), and only a few case series or scattered cases of antibody overlap have been reported in the literature, with some patients having an extremely poor prognosis. However, the mechanisms by which antibody overlap exists and its clinical significance are unclear. To improve our understanding of the clinical significance, treatment, and prognosis of AE antibody superposition, we conducted a retrospective analysis of the clinical characteristics, treatment, and prognostic details of 22 patients with AE who were treated at multiple clinical centers in China.

## Materials and methods

### Patients

We retrospectively identified 276 patients who were examined between January 2016 and June 2021 with a definite diagnosis of AE, according to the diagnostic criteria suggested by Graus et al. (6). Data were collected from four clinical centers (Qilu Hospital of Shandong University; The First Affiliated Hospital of Shandong First Medical University; Liaocheng People’s Hospital; and Affiliated Hospital of Binzhou Medical College) in Shandong, East China.

The inclusion criteria were as follows: (i) confirmed AE, (ii) serum and/or cerebrospinal fluid (CSF) that tested positive for one or more positive anti-neuronal antibodies, and (iii) reasonable exclusion of other disorders. The exclusion criteria were as follows: (i) serum and/or CSF that tested positive for only one positive anti-neuronal antibody and (ii) patient loss to follow-up. This study was approved by the Ethics Committees of the Qilu Hospital of Shandong University (approval number: KYLL-202008-044). All the patients or their families provided written informed consent.

### Coexisting autoantibodies testing

Serum and CSF samples from all 276 patients were sent to the same testing center for evaluation. Autoantibodies to Hu, Yo, Ri, Amphiphysin, SOX1, GAD65, CV2, and Ma2 were tested by using indirect immunofluorescence testing(tissue-based assay) (Euroimmun, Lubeck, Germany)and verified by Western blot. Autoantibodies to NMDAR, GABABR, AMPAR, CASPR2, LGI1,mGLuR5, glial fibrillary acidic protein (GFAP), AQP4, myelin oligodendrocyte glycoprotein were assessed (MOG) *via* an indirect immunofluorescence protocol with both cell-based assay and tissue-based assay.

### Data collection and follow-up

Clinical manifestations, serum, CSF, imaging, electroencephalography (EEG), and other auxiliary examination results and treatment information of patients with antibody coexistence were collected. We used modified Rankin Scale (mRS) scores to assess the effects of treatment and outcomes during the follow-up. Patients were followed-up every 2–3 months throughout the first year post-discharge and every 6 months thereafter. Patients with an mRS score of ≤2 were defined as having a “good prognosis,” and those with an mRS score of >2 were defined as having a “poor prognosis.”

### Statistical analysis

Statistical analyses were performed using GraphPad Prism software version 8.0 (GraphPad Software, San Diego, CA, USA) and SPSS version 26.0 (IBM Corp, Armonk, NY, USA). Summary statistics were reported as medians (range, minimum–maximum) for the continuous variables and as frequencies and percentages for the categorical variables.

## Results

### General demographic characteristics and coexistence of AE-related antibodies

**Figure 1:**
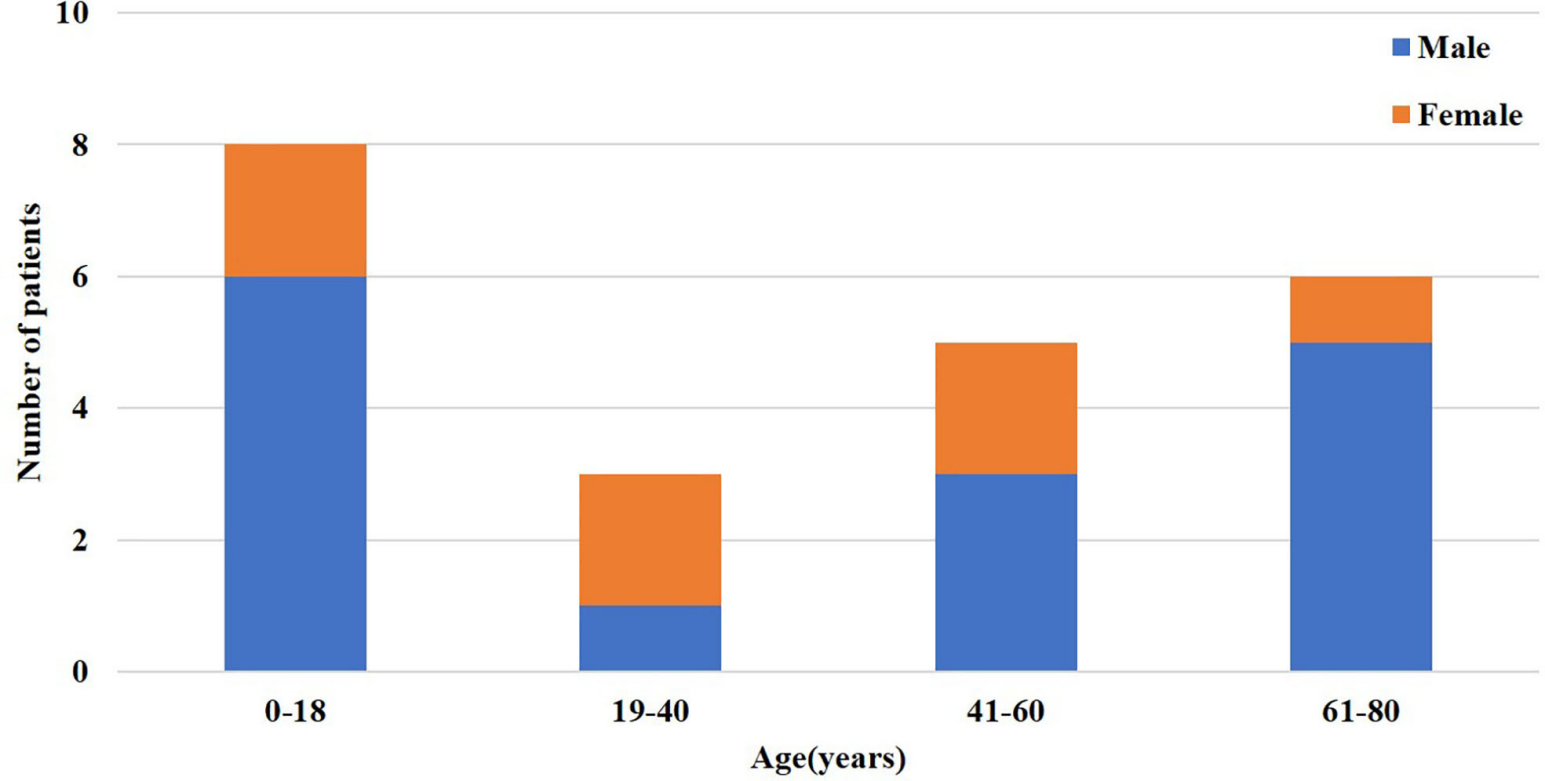
Distribution of sex and age of patients with the coexistence of multiple anti-neuronal antibodies.

**Figure 2:**
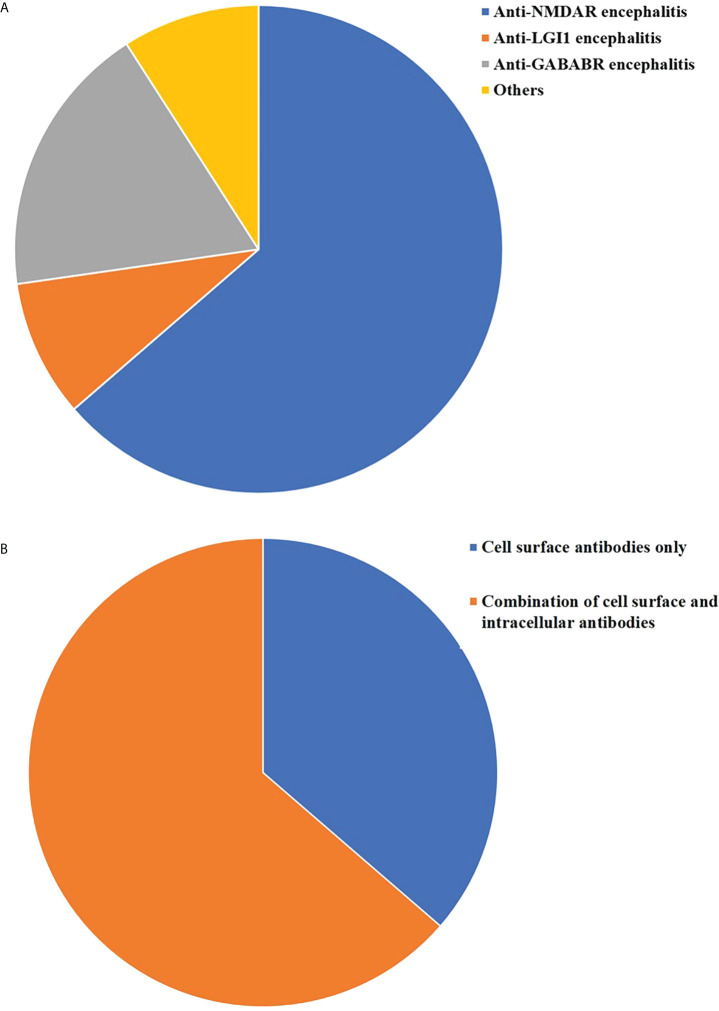
Distribution of overlapping anti-neuronal antibodies. **(A)** Proportion of different types of multiple anti-neuronal antibodies in 22 patients **(B)** Distribution of different types of anti-neuronal types in 22 patients.

Among the 276 antibody-positive AE patients identified *via* electronic medical record, 254 (92.03%) were single antibody positive, and 22 (7.97%) had complicated cases with two or more antibodies. The multi-antibody cohort included 7 females (31.82%) and 15 males (68.18%). In the anti-NMDAR antibodies group, 10.29% (14/136) of patients overlapped with other antibodies. Overlap with other anti-neuronal antibodies was observed in 2.25% (2/89) and 15.38% (4/26) of patients in the anti-LGI1 and anti-GABABR antibodies groups, respectively. In the multi-antibody cohort, 36.36% (8/22) of patients were younger than 18 years of age, and 50.0% (11/22) of the patients were between 41 and 80 years of age (**Figure 1**). The 22 cases were divided into two types: combination of antibodies against intracellular antigens and cell-surface antigens and antibodies against cell-surface antigens only. Among them, 14 patients (63.63%) were combined of cell surface and intracellular antibody, and the remaining 8 patients (36.36%) were detected to be cell surface antibody positive only(**Figure 2B**). Among them, 63.63% (14/22) of patients who were positive for anti-NMDAR antibodies, also had other types of AE-related antibodies, four anti-GABABR antibody-positive patients had other types of antibodies, and two anti-LGI1 antibody-positive patients had other types of antibodies (**Figure 2A**). The remaining three coexisting antibodies were one of CASPR2 and Ma2/Ta, one of MOG and GFAP. The details are listed in **Table 1**.

**Table 1:** Clinical characteristics of patients with co−existence of multiple anti−neuronal antibodies.

	Patient No./ Sex/Age (years)	Co-existent antibodies	Antibody findings		mRs at onset	Presentation and main symptoms	Complicated with tumors	Maximum mRS during AE	Immunotherapy	Relapse (Yes/No; recurrence interval, months)	mRs at last follow-up	Follow-up (months)
			CSF antibody titer	Serum antibody titer								
Combination of antibodies against intracellular antigens and cell-surface antigens	1/F/62	NMDAR, CV2	NMDAR(+);CV2(-)	NMDAR(+);CV2(+)	3	Impaired memory, unsteady walking	No	4	Steroids, IVIG, MMF	No	2	48
	2/M/10	NMDAR, CV2	NMDAR(+);CV2(+)	NMDAR(+);CV2(-)	2	Speech impairment, impaired memory, seizures	No	2	Steroids, IVIG	No	1	13
	3/M/46	LGI1, Yo	LGI1(+), Yo(-)	LGI1(+), Yo(+)	1	Abnormal sensation in the right lower extremity and seizures	No	2	Steroids, IVIG	No	1	30
	4/M/69	GABAB, Hu	GABAB(+), Hu(-)	GABAB(+), Hu(+)	3	Impaired memory, unresponsiveness, seizures, decreased level of consciousness	small-cell lung cancer	4	Steroids, IVIG	No	4	20
	5/F/60	GABAB, Yo, Hu	GABAB(+), Yo(-), Hu(-)	GABAB(+), Yo(+), Hu(+)	4	Memory impairment, abnormal mental behavior, seizures, generalized weakness	small-cell lung cancer	4	Reject	No	5	5
	6/M/46	NMDAR, GAD65, SOX1	NMDAR(+), GAD65(+), SOX1(+)	NMDAR(-), GAD65(+), SOX1(+)	3	Memory impairment, abnormal mental behavior, delusions of victimization, numbness in the right upper extremity	small-cell lung cancer	4	No immunotherapy	No	3	44
	7/F/59	CASPR2, Ma2	CASPR2(-), Ma2/Ta(-)	CASPR2(+), Ma2/Ta(+)	2	Sleep disorders, seizures, memory impairment	No	3	Steroids, IVIG	No	1	18
	8/M/25	NMDAR, AMPA1, AMPA2, Ma2	NMDAR(+), AMPA1(+), AMPA2(+),Ma2/Ta (-)	NMDAR(+), AMPA1(+), AMPA2(+),Ma2/Ta (+)	2	Memory impairment, psychomotor seizures, absence of sweating on the left side of the neck and face, vertigo, sleep disturbance	No	3	Steroids, IVIG	Yes, 6	1	8
	9/M/73	GABAB, Hu	GABAB(+), Hu(+)	GABAB(+), Hu(+)	4	Fever, dizziness, headache, unsteady walking, involuntary shaking of hands, hallucinations, hallucinations, abnormal mental behavior, decreased level of consciousness	mediastinal tumors	4	Steroids, IVIG	No	2	2
	10/M/70	GABAB, AMPAR1, SOX1	GABAB(+), AMPAR1(+), SOX1(-)	GABAB(+), AMPAR1(+), SOX1(+)	3	Memory loss, cognitive decline, not recognizing family members, delirium	small-cell lung cancer	4	Steroids, IVIG	No	5	2
	11/M/18	NMDAR, Yo	NMDAR(+), Yo(-)	NMDAR(+), Yo(+)	2	Fever, abnormal mental behavior, delirium	No	4	Steroids, IVIG, Plasma replacement therapy, Rituximab	No	3	4
	12/F/71	NMDAR, Yo	NMDAR(+), Yo(-)	NMDAR(+), Yo(+)	5	Dizziness, left lower extremity weakness with pain, inability to walk, seizures, babbling, decreased level of consciousness	No	5	Steroids, IVIG	No	3	52
	13/M/4	NMDAR, Ma2	NMDAR(+), Ma2/Ta(-)	NMDAR(+), Ma2/Ta(+)	3	Seizures, personality changes, decreased level of consciousness	No	4	Steroids, IVIG	No	1	48
	14/M/48	LGI1, Yo	LGI1(+), Yo(-)	LGI1(+), Yo(+)	3	Memory loss, cognitive decline, intermittent hallucinations, personality changes, seizures	No	4	Steroids, IVIG	No	2	3
Antibodies against cell-surface antigens only	15/M/17	NMDAR, CASPR2	NMDAR(+);CASPR2(+)	NMDAR(+);CASPR2(+)	3	Headache, seizures, numbness in right limb	No	4	Steroids, IVIG	No	2	23
	16/F/14	NMDAR, CASPR2	NMDAR(+);CASPR2(-)	NMDAR(+);CASPR2(+)	4	Fever, headache, impaired memory, seizures, impaired consciousness, coma	No	5	Steroids, IVIG	No	4	16
	17/M/14	NMDAR, CASPR2	NMDAR(+);CASPR2(-)	NMDAR(+);CASPR2(+)	3	Fever, diarrhea, headache, memory loss, pain in both lower limbs, paroxysmal involuntary movements of both lower limbs	No	3	Steroids, IVIG	No	2	21
	18/M/64	NMDAR, LGI1	NMDAR(+), LGI1(_+_)	NMDAR(+), LGI1(_+_)	4	Memory impairment, seizures, FBDS	No	4	Steroids, IVIG	Yes, 12	2	34
	19/F/5	NMDAR, MOG	NMDAR(+), MOG(+)	NMDAR(+), MOG(+)	3	Fever, diarrhea, left-sided limb weakness, visual impairment	No	4	Steroids, IVIG, MMF	No	1	15
	20/F/22	NMDAR, AMPAR1	NMDAR(+), AMPAR1(+)	NMDAR(+), AMPAR1(-)	3	Fever, headache, memory impairment, emotional irritability, involuntary movements of the jaw	No	4	Steroids, IVIG	No	4	22
	21/M/4	MOG, GFAP	MOG(+), GFAP(-)	MOG(+), GFAP(+)	2	Blurred vision and tearing in both eyes	No	2	Steroids, IVIG	No	1	8
	22/F/30	NMDAR, GFAP	NMDAR(+), GFAP(+)	NMDAR(-), GFAP(-)	4	Fever, lethargy, abnormal mental behavior, seizures, hitting and swearing, involuntary mouth and face movements, intermittent mouth chewing movements	No	4	Steroids, IVIG	No	1	4

AE, autoimmune encephalitis; F, female; M, male; Age, Age at onset of autoimmune encephalitis; CSF, cerebrospinal fluid.

Recurrence Interval, Time since symptoms relieved to relapse episode(months); FBDS, faciobrachial dystonic seizure; IVIG, intravenous immunoglobulins, 0.4 g/kg daily; MMF, mycophenolate mofetil.

### Clinical characteristics of patients with coexistence of antibodies

**Figure 3:**
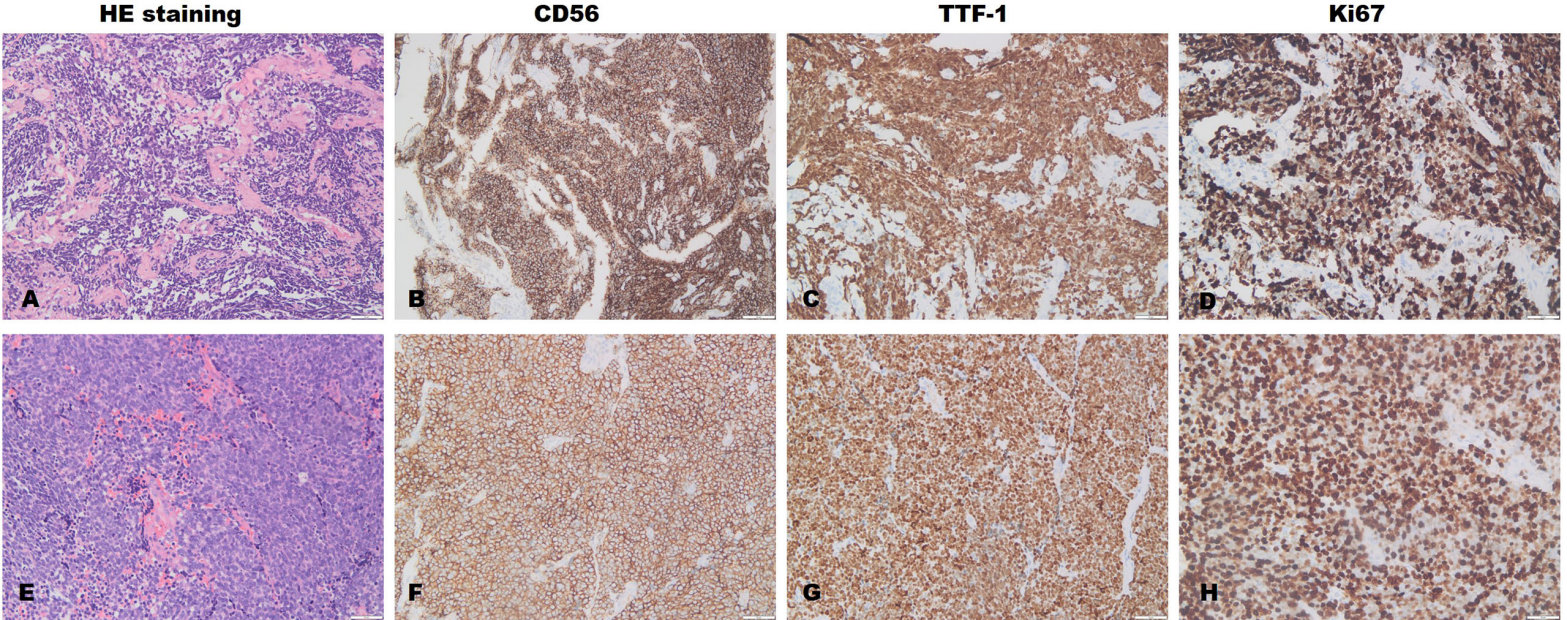
Lung **(A–D)** and lymphatic node **(E–H)** pathological examination. The pathological results of the lung of Patient 6 suggest small-cell lung cancer. Immunohistochemical results: The positive rates of CK (+), TTF-1 (+), syn (+), CD56 (+), CGA (-), CK7 (-), CK5/6 (-), and P40 (-); the positive rate of Ki-67 was approximately 80%. The pathological results of the right cervical lymph nodes in Patient 5 were indicative of metastatic small-cell carcinoma of the lung. Immunohistochemical results: The positive rates of CK punctate weak (+), CK7 (-), TTF-1 (+), syn (+), CGA punctate weak (+), CD56 (+), p63 (-); the positive rate of Ki-67 was approximately 85%.

The main symptoms of the 22 patients in this cohort included fever, dizziness, seizures, memory impairment, cognitive decline, sleep disorders, involuntary movements, and dyskinesia. The median mRS score at onset was 3 (range, 1–5), and the maximum median mRS score during the course of the disease was 4 (range, 2–5). Among the 22 patients, 5 (22.73%) patients had tumors; of these four had lung tumors and one had mediastinal tumors. Three of these five patients underwent pathological examination. The pathological examination confirmed the diagnosis of small cell lung cancer and malignant tumor of the lymph node (**Figure 3**).

CSF test results showed that the number of leukocytes increased in seven patients, of whom five patients showed lymphocytic reactions. The CSF pressure in most patients was normal or slightly elevated, and the patients’ levels of glucose, chloride, and protein were normal. Serum test results showed that 59.09% (13/22) of patients had other immune system antibodies. Among them, TG antibody(Ab) and anti-TPOAb appear to be more common (**Table 2**).

**Table 2:** Auxiliary findings of patients with co-existence of multiple anti-neuronal antibodies.

	Patient No./ Sex/Age (years)	Co-existent antibodies	CSF findings					Serum findings	EEG abnormal findings	MRI findings	18F-FDG PET/CT	Pathological findings
			Opening pressure (mmH_2_O)	WBC (×10^6^/L,0-6)	Glucose (mmol/L, 2.5-4.5)	Chloride (mmol/L, 120-130)	Protein (g/L, 0.15-0.45)				
Combination of antibodies against intracellular antigens and cell-surface antigens	1/F/62	NMDAR, CV2	80	4	3.26	124	1.16	Anti-cardiolipin antibody IgM(+)	Non-specific slow waves, pronounced in the frontotemporal region	Normal	NA	NA
	2/M/10	NMDAR, CV2	NA	2	3.77	127	0.22	Abnormal liver function	Diffuse slow waves in the left cerebral hemisphere with epileptiform discharges in the left central and middle temporal regions	Normal	NA	NA
	3/M/46	LGI1, Yo	170	1	4.12	123	0.39	TGAb(+)	Non-specific slow wave	Normal	NA	NA
	4/M/69	GABAB, Hu	80	6	5.8	126	0.62	Hyponatremia, hypokalemia, hypochlorhydria	Slow waves in frontotemporal region, spiny-slow complex waves in parieto-occipital region	Multiple foci of ischemia in the brain	Malignant lesion in the lower lobe of the right lung with possible regional lymph node metastasis. No significant abnormalities were found in brain.	NA
	5/F/60	GABAB, Yo, Hu	NA	NA	NA	NA	NA	TGAb(+), anti-TPOAb(+);ProGRP, 4957.45; NSE, 35.85	Diffuse slow waves with epileptiform discharges in the left temporal region	Normal	NA	Right cervical lymph node: Metastatic small cell lung cancer. Ki-67 positivity rate: 85%.
	6/M/46	NMDAR, GAD65, SOX1	190	4	5.22	126	0.38	ProGRP,101.59; NSE,24.7;	NA	Intracranial multiple demyelination	NA	Left lung tissue: small cell lung cancer of the lung. Ki-67 positivity rate: 80%.
	7/F/59	CASPR2, Ma2	125	5	2.90	128	0.31	CA-724, 8.36	Increased slow waves in frontotemporal region, spikes and spikes in both temporal regions	NA	NA	NA
	8/M/25	NMDAR, AMPA1, AMPA2, Ma2	175	6	4.9	130	0.25	TGAb(+), anti-TPOAb(+); Varicella zoster IgG(+); Anti-EB virus capsid antigen IgG and anti-EB virus nuclear antigen(+); Rubella virus IgG(+); Cytomegalovirus IgG(+);	More slow wave activity in the anterior head	Abnormal signals in the left frontal lobe and right insula	NA	NA
	9/M/73	GABAB, Hu	130	4	4.4	128	0.34	TGAb(+), anti-TPOAb(+); ProGRP, 281.8; CA-724,56.4; NSE,17.8; ANCA(+); APA lgG and lgM(+)	Abnormal	Multiple foci of ischemia in brain	High uptake of FDG in mediastinal lymph nodes, and no significant abnormalities in brain to suprafemoral segment	Malignant tumor of lymph node
	10/M/70	GABAB, AMPAR1, SOX1	NA	NA	NA	103	0.25	TGAb(+), anti-TPOAb(+);	NA	Abnormal signals in the right hippocampus	NA	lung cancer
	11/M/18	NMDAR, Yo	NA	252, lymphocyte reaction	Normal	Normal	Normal	Anti-Ro antibody(+); AMA M2(+); ANA(+)	NA	NA	NA	NA
	12/F/71	NMDAR, Yo	100	32, lymphocyte reaction	4.46	147	0.66	TGAb(+), anti-TPOAb(+);FT3 2.21; Abnormal liver function	NA	Multiple abnormal signals in the bilateral frontoparietal corticomedullary junction area	NA	NA
	13/M/4	NMDAR, Ma2	107	82, lymphocyte reaction	3.13	129	0.16	NA	Slow background rhythm	Normal	NA	NA
	14/M/48	LGI1, Yo	NA	NA	NA	112	NA	NA	Increased slow wave activity in bilateral frontal, central parietal, and anterior temporal regions	Abnormal signals in the right hippocampus	NA	NA
Antibodies against cell-surface antigens only	15/M/17	NMDAR, CASPR2	150	10	3.84	122	0.39	Elevated serum ammonia	Widespread low to moderate amplitude slow waves, pronounced in the frontotemporal region	Normal	NA	NA
	16/F/14	NMDAR, CASPR2	265	42	3.46	128	0.19	Elevated serum ammonia; serum rubella virus, cytomegalovirus IgG, and herpes simplex virus IgG (+)	Normal	Normal	NA	NA
	17/M/14	NMDAR, CASPR2	200	1	3.81	130	0.29	Serum anti-EBV capsid antigen IgM, anti-EBV capsid antigen IgG, and EBV nuclear antigen IgG(+).	Normal	White matter lesions in the bilateral cerebral hemispheres	NA	NA
	18/M/64	NMDAR, LGI1	185	7, lymphocytes, 82%	4.18	107	0.29	Hyponatremia, hypochlorhydria, TGAb(+), anti-TPOAb(+)	Increased diffuse slow wave activity	Ischemic focus in the right frontal lobe	Significantly lower FDG metabolism levels in multiple left cranial regions (left parietal, occipital, parahippocampal gyrus and dorsal thalamus)	NA
	19/F/5	NMDAR, MOG	NA	4	4.04	128	0.24	NA	NA	Multiple demyelinating lesions in white matter of left cerebral hemisphere and bilateral optic nerve demyelinating lesions	NA	NA
	20/F/22	NMDAR, AMPAR1	190	14, lymphocyte reaction	4.96	130	0.26	TGAb(+), anti-TPOAb(+);Rubella virus IgG, cytomegalovirus IgG and herpes simplex virus types I and II IgG (+)	Normal	Abnormal signals in bilateral temporal lobe, mesencephalon, left hippocampus, midbrain and pontine brain	NA	NA
	21/M/4	MOG, GFAP	NA	2	4.84	131	0.12	NA	NA	Multiple abnormal signals in brain and cervical medulla, with bilateral inflammatory changes in the optic nerve	NA	NA
	22/F/30	NMDAR, GFAP	140	20	5.13	130	0.07	Abnormal liver function	Abnormal	Normal	NA	NA

ALT, glutamic pyruvic transaminase; AST, glutamic oxaloacetic transaminase; CA-724, carbohydrate antigen,U/ml.

ProGRP, pro-gastrin-releasing peptide, pg/ml; NSE, neuron-specific enolase, ng/ml.

TGAb, anti-thyroglobulin antibodies; anti-TPOAb, anti-thyroid peroxidase autoantibody; anti-Ro antibody(+); AMA M2(+); ANA(+); FT3, free triiodothyronine, pmol/L.

ANCA, anti-neutrophil cytoplasmic antibodies; APA, antiphospholipid antibody.

AE, autoimmune encephalitis; F, female; M, male; Age, Age at onset of autoimmune encephalitis; NA, not applicable or available; CSF, cerebrospinal fluid.

Recurrence Interval, Time since symptoms relieved to relapse episode(months); FBDS, faciobrachial dystonic seizure.

NA, not available.

**Figure 4:**
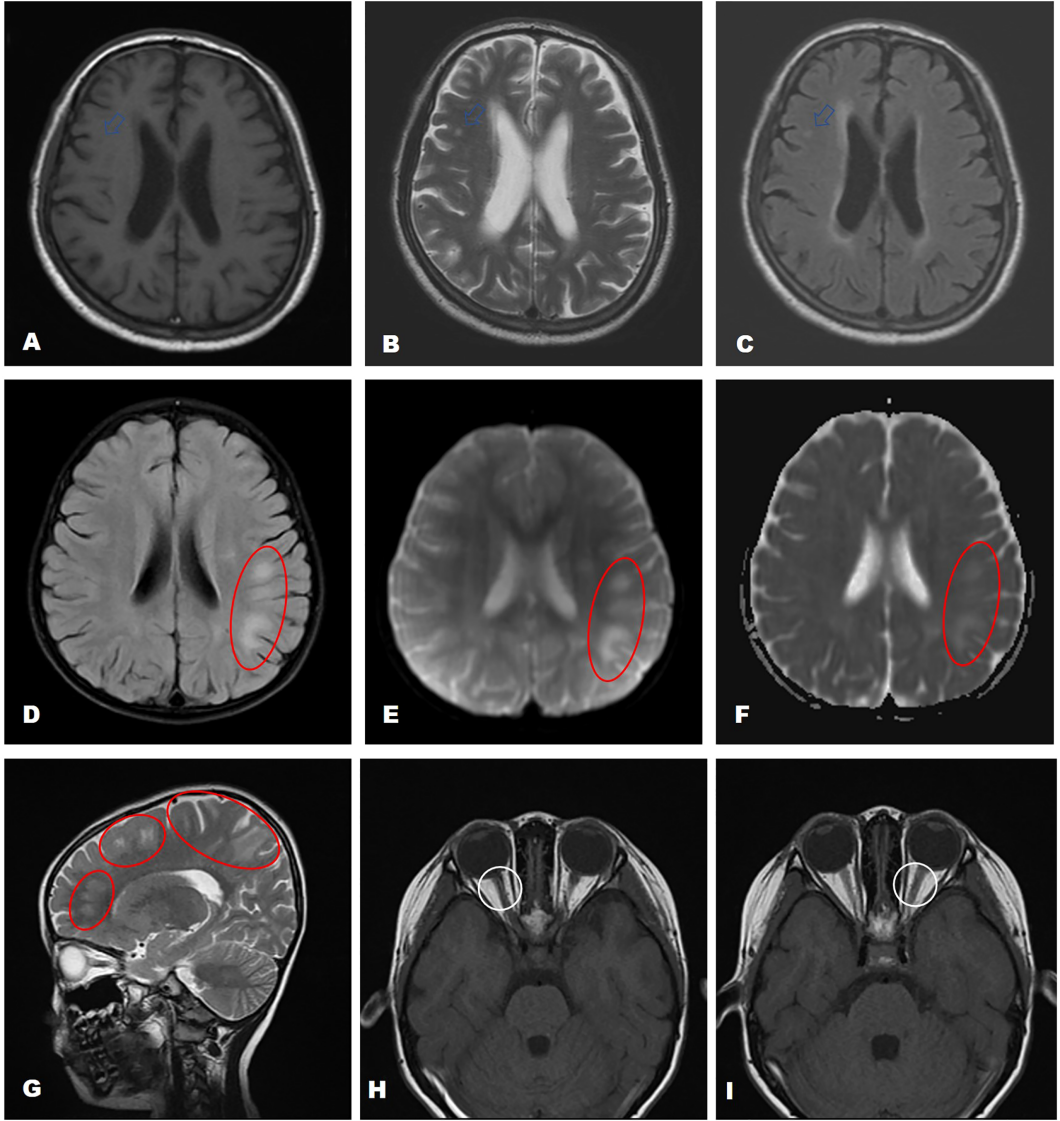
MRI findings of AE patients with coexisting antibodies. The brain MRI of Patient 18 showed a small patchy area with long T1 and long T2 signal shadows in the right frontal lobe with a high T2-FLAIR signal (**A–C**, marked by a red circle and a white arrow). Patient 19 showed multiple demyelinating lesions in the white matter of the left cerebral hemisphere (**D–G**, marked by a red circle) and the bilateral optic nerve **H**, **I** Fat suppression-T2 sequence).

**Figure 5:**
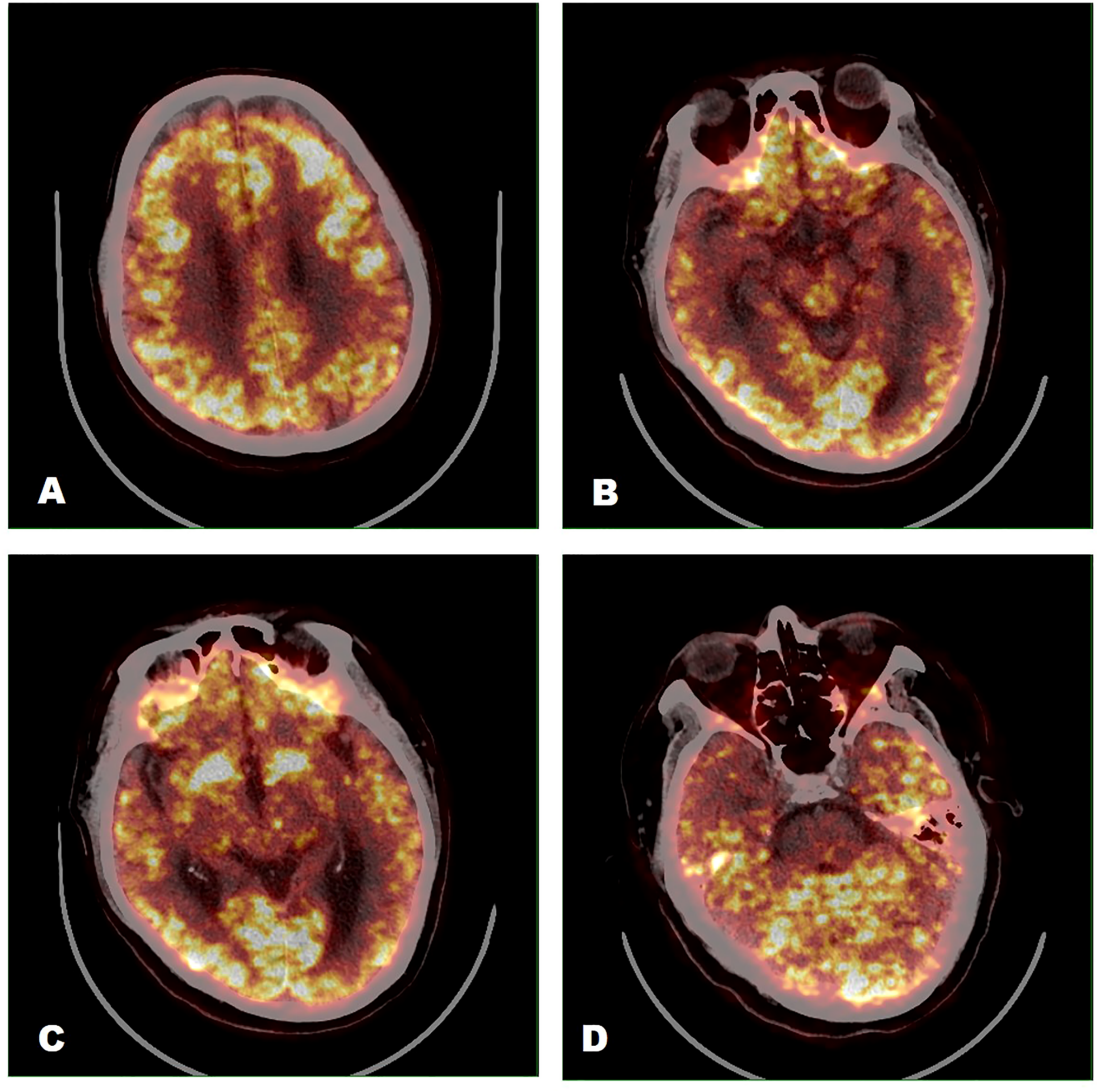
^18^F-FDG PET/CT image findings (Patient 18). Significantly lower FDG metadata levels were observed in the left parietal **(A)**, occipital **(C)**, parahippocampal gyrus **(B, D)**, and dorsal thalamus **(C)**.

The patients’ EEG scans primarily showed focal nonspecific slow waves, with only one patient (Patient 7) showing spikes in both temporal regions. Twelve patients showed abnormal brain magnetic resonance imaging (MRI) signals, of whom three underwent ^18^F-fluorodeoxyglucose mediated positron emission tomography/computed tomography (18F-FDG PET/CT) scans. MRI scans of Patient 19 showed multiple demyelinating lesions in the white matter of the left cerebral hemisphere and the bilateral optic nerve (**Figures 4D–I**). There appeared to be a discrepancy in the location of the lesions, as shown by brain MRI and PET scans. Among them, the brain MRI scan of Patient 18 showed an ischemic focus in the right frontal lobe (**Figures 4A–C**), while the 18F-FDG PET/CT scan showed significantly lower FDG metadata levels in multiple left cranial regions (left parietal, occipital, para-hippocampal gyrus, and dorsal thalamus) (**Figure 5**). The brain MRI of Patients 4 and 9 showed multiple foci of ischemia; however, no significant abnormalities were observed in the brain PET scans. Additional details are listed in **Table 2**.

### Treatment and prognosis of AE patients with coexisting antibodies

In total, 20 patients were treated with steroids and intravenous immunoglobulin, of whom 18 showed varying degrees of symptomatic improvement after the administration of first-line immunotherapy. Two patients (Patients 11 and 19) had a poor response to the first-line immunotherapy and needed second-line treatment. Patients 8 and 18 developed relapses at 6 and 12 months after discharge, respectively. Three patients in our retrospective study expired. Patients 4, 5, and 10 died of tumor progression or complications of chemotherapy at 20, 5, and 2 months after discharge, respectively (**Table 1**).

### Clinical characteristics of patients with single anti-neuronal antibodies

Among the 254 AE patients with a single-positive anti-neuronal antibody, there were 122 cases of NMDAR, 187 cases of LGI1, 22 cases of GABABR, 9 cases of CASPR2, and 14 other subtypes AE (2 cases of AMPA1, DPPX, and 3 cases of Amphiphysin; 1 case of AMPAR (type 2), CV2, Hu, Yo, CV2, Ri, GDA65, and mGLuR5). We also analyzed the chief clinical characteristics of patients in the single antibody cohort. There were 145 (57.08%) male and 104 (42.91%) female patients. The median age of onset of these patients was 38 years (range, 1–80 years), and the median mRS at onset was 3 (IQR, 3–4). In this cohort, the common clinical manifestations of patients were seizures (70.47%), memory deficit (64.96%), mental behavioral disorders (57.87%), disturbance of consciousness (39.37%), and movement disorders (29.92%). The common symptoms of different subtypes of AE are different. Notably, 14 (5.51%) patients with single antibody-positive AE were admitted to the ICU for serious illness. Among the 254 patients with AE, 23 (9.09%) had concomitant tumors. Anti-NMDAR encephalitis was predominant with teratoma, whereas lung tumors were more frequent in the anti-GABABR encephalitis cohort. No tumors were detected in both the anti-LGI1 encephalitis and anti-CASPR2 encephalitis groups. Overall, 25 (9.84%) patients experienced recurrence, including 16 patients with anti-NMDAR encephalitis, 6 anti-LGI1encephalitis, 1 anti-GABABR encephalitis, 1 anti-CASPR2 encephalitis, and 1 anti-AMPAR (type 2) encephalitis. Details are presented in **Table 3**.

**Table 3:** Clinical characteristics of patients with single anti-neuronal antibodies (n=254).

Characteristics	Total(n=254)	Anti- NMDAR encephalitis(n=122)	Anti-LGI1 encephalitis(n=87)	Anti-GABABR encephalitis(n=22)	Anti- CASPR2 encephalitis(n=9)	Other subtypes AE(n=14)
Sex, Male/Female	145/104	54/68	63/24	19/3	6/3	8/6
Age at onset, y; median, range	38 (1–80)	18 (1–80)	59 (5–77)	65 (17–77)	27 (3–45)	24 (2–79)
mRS at onset; median, IQR	3 (3–4)	3 (3–4)	3 (2–3)	3 (3–4)	3(2-4)	3 (3–4)
Presentation and main symptoms, n (%)	Seizures, 179 (70.47%)Memory deficit, 165 (64.96%)Mental behavioral disorders, 147 (57.87%)Disturbance of consciousness, 100 (39.37%)Movement disorder, 76 (29.92%)	Mental behavioral disorders, 97 (79.51%)Seizures, 95 (77.87%)Memory deficit, 71 (58.20%)Disturbance of consciousness, 69 (56.56%)	Memory deficit, 65 (74.71%)Seizures, 58 (66.67%)Mental behavioral disorders, 26 (29.89%)Disturbance of consciousness, 13 (14.94%)	Memory deficit, 19 (86.36%)Seizures, 17 (77.27%)Disturbance of consciousness, 15 (68.18%)Mental behavioral disorders, 14 (63.64%)	Mental behavioral disorders, 4 (44.44%)Seizures, 3 (33.33%)Memory deficit, 2 (22.22%)Movement disorder, 2 (22.22%)	Memory deficit, 8 (61.54%)Mental behavioral disorders, 6 (46.15%)Seizures, 6 (46.15%)Disturbance of consciousness, 2 (15.38%)
Admission to ICU	14 (5.51%)	10 (8.20%)	2 (2.30%)	2 (9.09%)	0	0
Complicated with tumors	23 (9.09%)	13 (10.66%); Teratoma (n=12), Lung tumor (n=1)	0	8 (36.36%); Glioma (n=1), Mediastinal tumor (n=2), Lung tumor (n=5)	0	2 (15.38%); Lung tumor (n=1, anti-Hu Ab+), lymphoma (n=1, anti-Yo Ab+)
Relapse	25 (9.84%)	16 (13.11%)	6 (6.90%)	1 (4.54%)	1 (11.11%)	1 (7.69%)

## Discussion

We described the clinical characteristics, treatment, and prognosis of 22 patients with coexisting neuronal antibodies, and observed that the clinical symptoms and laboratory findings of patients with coexisting antibodies were more diverse and atypical at onset compared with single-antibody positive AE, leading to increased difficulty in early diagnosis and treatment. Our study provided new evidence for the diagnosis and prognosis of multiple antibody-positive patients with AE. To our knowledge, this was the first case series report on multiple-antibody coexistence in patients with AE from multiple clinical centers in the East of China.

The coexistence of multiple antibodies in AE is uncommon. Currently, studies in China and abroad are predominantly small-sample case series and single-case reports (10, 12–14). In 2018, Fan et al., in a study comprising 42 patients with MOG-Ab disease and 491 patients with neuromyelitis optica spectrum disorder (NMOSD), reported that 11.9% of patients with MOG-Ab disease and 0.6% of patients with NMOSD had overlapping NMDAR-Ab (10). A study involving 189 patients with AE in Southwest China in 2019 showed that 8 (4.23%) patients had two co-existing neuronal antibodies, of whom 50% (4/8) of patients with anti-NMDAR antibody-positivity were observed to have co-existing antibodies of other types (11). In our cohort, there were only 22 (7.97%) patients with multiple antibodies, of whom 63.63% (14/22) of patients with anti-NMDAR antibody-positivity were observed as having other types of co-existing antibodies.

Our results were similar to those of previous reports. In addition to anti-NMDAR encephalitis, anti-GABABR encephalitis was more often associated with other anti-neuronal antibodies. In a study of 20 cases of anti-GABABR encephalitis by Höftberger et al. in 2013 (15), seven cases (35%) had additional antibodies, including three cases with SOX1, two amphiphysin, one GAD65, one NMDAR, and one Ri (ANNA2). In our study, 75% (3/4) of anti-GABABR antibody-positive patients had coexisting paraneoplastic antibodies such as Hu and Yo. In general, the positive phenomenon of multiple anti-neuronal antibodies in AE is rare, and it also suggests that multiple antibodies may coexist in the presence of NMDAR and GABABR antibody-related AE.

The presence of coexisting anti-neuronal antibodies may lead to diversification of the clinical characteristics of AE and the presence of atypical clinical and radiographic features (10, 16–18). For example, anti-NMDAR encephalitis may co-exist with a demyelinating disease in the presence of anti-MOG or anti-GFAP antibodies. In our study, patients with anti-AMPAR encephalitis were noted to also have stiff-person syndrome (anti-GAD) or sensory neuropathy (anti-amphiphysin). As reported by Höftberger in 2013, in a study of anti-GBABR encephalitis (15), patients with coexisting additional NMDAR antibodies showed prominent psychiatric symptoms, whereas patients with coexisting GAD65 antibodies developed refractory seizures. In our study, seizures, memory deficit, and mental behavioral disorders were more common in the single-antibody cohort at onset, whereas the clinical presentation of the multi-antibody cohort was more diverse. For example, Patient 14—who had four antibodies (NMDAR, AMPA1, AMPA2, and Ma2)—experienced vertigo, sleep disturbances, memory impairment, seizures, and absence of sweating on the left side of the neck and face; these symptoms were diverse and atypical. Patient 13 with NMDAR-Ab and MOG-Ab predominantly showed limb weakness and visual impairment and had multiple demyelinating lesions in the white matter of the left cerebral hemisphere and the optic nerve. Therefore, the potential for the coexistence of non-specific clinical symptoms associated with each anti-neural antibody, also suggests the importance and necessity of antibody testing, while it should be noted that whether there is a certain correlation between antibody overlap and phenotype overlap still needs more studies to clarify.

The clinical symptoms and laboratory findings of multiple antibody-associated AE may overlap with those of other autoimmune diseases and are more diverse and atypical than those of a single antibody-positive AE (4, 19–21). The results of tests for autoimmune antibodies are not readily available at some institutions and may take days or even weeks to become available. This makes the early diagnosis and treatment of multiple AE more difficult, particularly in some AE patients who are negative for anti-neuronal antibodies and whose initiation of immunotherapy is more dependent on clinical manifestations and ancillary findings during the course of the disease. Therefore, new biomarkers should be explored to assist in early diagnosis (22–24).

AE imaging has also received considerable attention in recent years. MRI findings in the brain have been more commonly reported in previous studies. The brain MRI in patients with AE involves most commonly the temporal lobe or hippocampus unilaterally or bilaterally and may sometimes accumulate in the thalamus, occipital lobe, and brainstem with or without enhancement. Previously, an ^18^F-FDG PET/CT scan was primarily used to assess whether the etiology of encephalitis was an occult malignancy (18, 25, 26). However, recent studies have begun to note the positive role of cerebral ^18^F-FDG PET/CT scans in the evaluation of patients with AE (27, 28). A retrospective study in 2017 assessed the positivity rate of MRI versus ^18^F-FDG PET/CT scans, which included 23 patients who were proven to have antibody-positive anti-NMDAR encephalitis, showed that a PET/CT scan more often showed abnormal findings compared with a brain MRI scan (16). In our study, the predominant finding from the brain ^18^F-FDG PET/CT imaging was lobar hypometabolism, with hypometabolism being observed most commonly in the parietal and occipital lobes. Another study involving 24 patients with anti-NMDAR encephalitis in the acute phase in 2021 showed the characteristics of patients with anti-NMDAR encephalitis of different etiologies (18). Those findings showed that patients in the cryptogenic and paraneoplastic subgroups exhibited hypermetabolism in the frontotemporal and basal ganglia and hypometabolism in the occipital lobe. Other patients, secondary to viral encephalitis, exhibited significantly lower metabolism in the bilateral occipital and temporal lobes on the side of viral encephalitis and part of the basal ganglia. This suggests that the brain ^18^F-FDG PET/CT scan may be more likely to be abnormal than the MRI scan of the brain and that patients with AE triggered by different factors present distinct cerebral metabolic patterns. In the preliminary exploratory stage, cumulative evidence supported the value of the characteristic presentation of ^18^F-FDG PET/CT scanning as an early diagnostic indicator of AE.

Patients with combined multiple anti-neuronal antibody autoimmunity, regardless of the clinical manifestations, have a more complex and diverse disease than those with AE with a single antibody; however, they are still treated primarily with immunotherapy (7, 29, 30). First-line immunotherapy included steroids, intravenous immunoglobulin, and plasma exchange, and second-line immunotherapy included options such as rituximab, mycophenolate mofetil, and cyclophosphamide. Early tumor resection or other antitumor therapy should be performed in the presence of a combined tumor (4, 30–32). The co-existence of different antibody stacks leads to differences in immunotherapy response and long-term prognosis. AE patients with multiple anti-neuronal antibodies, especially the paraneoplastic antibodies (e.g., Hu, Yo, and Ri), often have other tumors and have a poor response to immunotherapy and a poor prognosis. In a study of 17 cases of anti-GABABR encephalitis, 8 patients died in the last follow-up; seven of these eight patients died as a result of tumor progression or complications of chemotherapy (31). Among them, five patients had additional antibodies: three SOX1, one Amphiphysin, and one Ri (ANNA2). The only patient without a tumor had refractory status epilepticus, in association with antibodies to GAD65, and was the cause of death. They also observed that patients with small-cell lung cancer had a shorter survival time than patients without tumors.

In our study, 20 patients were treated with steroids and intravenous immunoglobulin, and most patients showed an improvement after first-line immunotherapy. Three patients died of tumor progression or complications of chemotherapy, and all of them had small-cell lung cancer. It is important to note that 22.73% of patients had tumors in the multi-antibody cohort, while 9.09% of patients with single antibody positive had concomitant tumors. Furthermore, in the multi-antibody cohort, four (100%) patients with GABABR antibody (+) had tumors (small-cell lung cancer being more common), whereas 36.36% of patients with anti-GABABR encephalitis had tumors in the single-antibody positive group. This suggests that when these AE-associated antibodies coexist in superposition, they are highly suggestive of tumors. Given the poor prognosis of AE combined with tumors, especially with small-cell lung cancer, early tumor screening is of great significance. Particularly in AE patients with combined GABABR and paraneoplastic antibodies, regular tumor screening and long-term follow-up are necessary if the tumor is not detected early in the course of the disease.

This study has certain limitations. Although a more varied demographic background is implied by this being a multicenter study, to some extent this study reflects the characteristics of patients with multi-antibody AE, specifically in eastern China; the geographical limitations of the regional design limit the variability of the demographic characteristics, thus limiting our ability to generalize the findings. The current study is retrospective and has limitations in terms of completeness of patient data and monitoring of dynamic changes in the disease. In addition, another limitation of this study is that most patients did not retest autoimmune encephalitis related antibodies during and after treatment, so the possibility of false positives for some antibodies cannot be excluded. On the one hand, we need to objectively assess the clinical phenomenon and significance of the coexistence of multiple antibodies, on the other hand, dynamic detection of antibodies is required to exclude false positives. Thus, large-sample, multi-center, prospective, and longitudinal studies are needed.

## Conclusion

The coexistence of multiple anti-neuronal antibodies is rare, and the superposition of antibody types may lead to superimposed or atypical clinical features, making early diagnosis difficult. Antibody superimpositions respond differently to immunotherapy, and differences in long-term prognosis exist. The coexistence of multiple anti-neuronal antibodies, especially when combined with paraneoplastic antibodies, suggests the possibility of a combined potential tumor range. The clinical significance and mechanism of the coexistence of multiple anti-neuronal antibodies still need to be further studied.

## Data availability statement

The original contributions presented in the study are included in the article/supplementary material. Further inquiries can be directed to the corresponding author.

## Ethics statement

The studies involving human participants were reviewed and approved by the Ethics Committee of Qilu Hospital, Cheeloo College of Medicine, Shandong University. Written informed consent to participate in this study was provided by the participants’ legal guardian/next of kin.

## Author contributions

X-WL conceived the study and supervised this work. SQ conceived the study, organized and statistical data, and drafted the manuscript. S-CZ collected and organized datas. Z-HW, LW, R-RZ, H-YL, YJ, L-LL, M-LW, and A-HW assisted in collecting datas. All authors reviewed and approved the final manuscript. All authors contributed to the article and approved the submitted version.

## Funding

The National Natural Science Foundation (No. 81873786) supported this work.

## Conflict of interest

The authors declare that the research was conducted in the absence of any commercial or financial relationships that could be construed as a potential conflict of interest

## Publisher’s note

All claims expressed in this article are solely those of the authors and do not necessarily represent those of their affiliated organizations, or those of the publisher, the editors and the reviewers. Any product that may be evaluated in this article, or claim that may be made by its manufacturer, is not guaranteed or endorsed by the publisher.
